# Methodological and Short-Term Diurnal Variation in Surface and Cargo Proteins in Plasma Extracellular Vesicles

**DOI:** 10.3390/cimb48010120

**Published:** 2026-01-22

**Authors:** Hubert Krzyslak, Weronika Maria Szejniuk, Ursula Falkmer, Bent Honoré, Malene Møller Jørgensen, Charlotte Sten, Shona Pedersen, Gunna Christiansen, Søren Risom Kristensen

**Affiliations:** 1 Department of Clinical Biochemistry, Aalborg University Hospital, 9000 Aalborg, Denmark; 2 Department of Oncology, Aalborg University Hospital, 9000 Aalborg, Denmark; 3 Department of Clinical Medicine, Aalborg University, 9000 Aalborg, Denmark; 4 Department of Biomedicine, Aarhus University, 8000 Aarhus, Denmark; 5 Department of Clinical Immunology, Aalborg University Hospital, 9000 Aalborg, Denmark; 6 Department of Basic Sciences, Qatar University, Doha P.O. Box 2713, Qatar; 7 Department of Health Science and Technology, Aalborg University, 9000 Aalborg, Denmark

**Keywords:** extracellular vesicles, proteomic profiling, nanoparticle tracking analysis (NTA), biological variability, methodological variability, EV-Array

## Abstract

Extracellular vesicles (EVs) are known as potential biomarkers for several diseases; nevertheless, the degree of technical and biological variability is not yet adequately characterized. Because pre-analytical factors such as blood collection time and EV subpopulation could confound biomarker studies, we performed a pilot study systematically quantifying methodological and biological variability including EV-Array (surface proteins), and proteome characterization of cargo. Plasma samples from six healthy adults were collected at two time points (morning and afternoon) and plasma was analyzed with EV-Array, and isolated EVs were analyzed using nanoparticle tracking analysis (NTA), and label-free mass spectrometry (LC-MS/MS). Methodological repeatability was high for NTA particle size (3.3% CV) and LC-MS (8.2% CV), and lower for EV-Array surface markers (22.6% CV). Variations between samples were reasonable for NTA-size, EV-Array and LC-MS/MS (5–21%) and substantially lower than between-subject variation. No evidence of systemic morning–afternoon shifts in particle size and concentration or EV cargo was observed, although small effects cannot be excluded. The same was true for most surface markers, but minor but statistically significant reductions in a few specific surface markers occurred in afternoon EV-Array samples. In this pilot we therefore do not observe any major systemic diurnal bias in healthy individuals in samples collected a.m. vs. p.m. Despite the small sample size, this study underscores the importance of accounting for individual variability and methodological standardization when designing EV-based biomarker research.

## 1. Introduction

Extracellular vesicles (EVs) are a heterogeneous population of phospholipid bilayer-encased particles, often classified by their size and formation mechanism as small EVs (sEVs; 50–150 nm, predominantly exosomes) and large EVs (lEVs; 100–1000 nm, primarily microvesicles) [1]. They are recognized for their potential role in cellular communication and many diseases and malignancies [1,2,3,4,5,6]. EV cargo and surface proteins have been used as a source of disease biomarkers, as cargo proteins and other molecules are known to reflect the physiological state of the parent cell, while EV surface proteins hold a promise for rapid-access biomarkers due to their roles in cellular communication and disease-associated signaling pathways [7,8,9]. Still, the extent of normal biological variability in their cargo and surface proteins and their role in normal physiological processes remains poorly understood [5,10,11].

All methods have an inherent variation and some methods in EV research may have a sizeable variation. Variations in size, molecular composition, and cellular origin of the EVs limit the separation and identification of distinct EV subpopulations, challenging standardization and reproducibility across different studies [3,12,13,14,15,16,17]. Small variations in isolation protocols or analytical techniques can result in the enrichment of specific EV populations and can lead to heterogeneity in experimental outcomes limiting the comparability of data between laboratories [3,8,9,11]. In biomarker research, it is crucial to account for both preanalytical and analytical variability, as well as intra-individual fluctuations, to ensure repeatability [12,18,19]. While some evidence suggests a diurnal variation in EV secretion and abundance, it is still unclear if these oscillations affect the quantification of clinically important EV proteins [20,21]. Consequently, the use of complementary characterization approaches is essential for addressing EV heterogeneity and advancing the research towards reliable biomarker discovery and clinical application [3,8,9,15]. Prior work has quantified aspects of within- and between-subject EV variability in selected biological fluids and EV fractions, including serum sEV cargo and particle size/concentration (Newman et al.) and urinary EV proteomics (Oeyen et al.) [20,21]. In contrast, the present study provides an integrated, multi-platform variability assessment from sample to result in paired plasma EV fractions obtained by differential ultracentrifugation (lEV and sEV), combining particle metrics (NTA), surface phenotyping on plasma (EV-Array), and cargo proteomics (label-free LC-MS/MS). Thus far, there has been no such comprehensive characterization of the technical and biological sources of variance. The aim of this exploratory pilot study is, therefore, to thoroughly evaluate the technical and biological heterogeneity associated with EV measurements in healthy individuals. Specifically, we quantify (i) the variation in each method, i.e., variation in results from the same sample (CVmethod), (ii) the variation in two different samples (CVdupl) from the same time point, (iii) the variation in two samples from different time points, i.e., determine whether sampling within a clinically realistic daytime window (between 08:00 and 13:00 at two distinct time points) introduces systematic differences in EV readouts (called CV_am vs. pm_) which may confound biomarker studies and, finally (iv) the variation between subjects (CVbiological). The quantitation of variations is performed for particle size, particle concentration, surface proteins, and cargo proteins across the three analytical platforms.

## 2. Materials and Methods

### 2.1. Study Population

**Figure 1:**
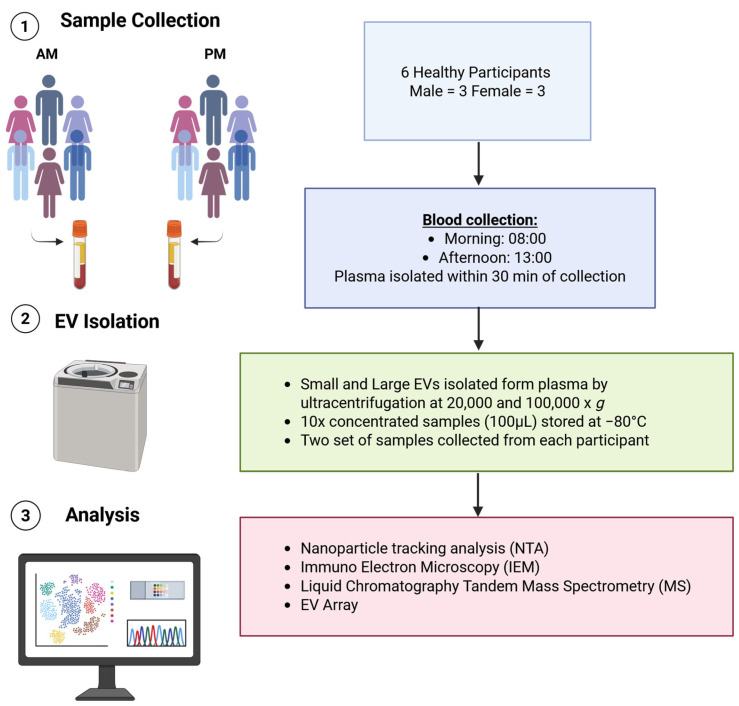
Schematic representation of the study design. Six healthy participants between 40 and 55 years old were recruited to elucidate EV variability. Blood was collected in the morning (AM) and afternoon (PM). Two sets of two 9 mL citrate plasma tubes were collected from each participant and used for the experiments. EVs were isolated by differential ultracentrifugation concentrated to the volume of 10–100 µL for downstream analysis. Nanoparticle tracking analysis was used to determine particle size and concentration, IEM was used to estimate the size and morphology of isolated EVs, mass spectrometry for EV protein cargo characterization and EV-Array for EV surface protein characterization. Created in BioRender. Krzyslak, H. (2026) https://BioRender.com/j02wtyb (accessed on 16 January 2026).

In this pilot study six healthy individuals (3 female, 3 male) were recruited from Aalborg, Denmark (Figure 1) between 1 and 28 June 2023. All participants were between 40 and 55 years old and did not receive any pharmacological treatment. This study was conducted according to the declaration of Helsinki and was approved by the ethical committee of Northern Jutland (N-20230011) on the 4th of May 2023. All study participants signed a written informed consent before the inclusion in the study. The mean age and BMI for the 3 males was 43 (41–46) and 25.0 (3.8), while for the 3 females it was 49 (40–55) and 20.4 (1.6), respectively.

### 2.2. Blood Sample Collection

Blood samples were obtained from the participants at two specific time points during the same day: in the morning between 8:00 and 8:30 a.m. (called AM), and in the afternoon between 12:30 and 1 p.m. (called PM). The blood was drawn from the antecubital vein using a 21-gauge needle attached to a vacutainer system (Vacuette, Greiner Bio-One, Kremsmünster, Austria). The blood was drawn in 9 mL tubes containing 0.105 M (3.2%) trisodium citrate (BD Vacutainer^®^, Berkshire, UK) as anticoagulant. To isolate Platelet Poor Plasma (PPP), the blood samples underwent a double centrifugation at 2500× *g* for 15 min each at room temperature. After centrifugation, the plasma was collected one cm above the buffy coat. The plasma samples were stored at −80 °C for subsequent analyses but at −70 °C for MS.

### 2.3. EV Isolation

The EV containing plasma was isolated by two-step centrifugation. Firstly, the lEVs (lEV-pellet) were isolated by centrifuging 1 mL of plasma at 20,000× *g* for 60 min at 4 °C using a Heraeus Multifuge 3S-R (Thermo Scientific, Waltham, MA, USA) with a #3332 fixed angle rotor (Thermo Scientific, USA). The sEV pellet was isolated using an Avanti J-30i centrifuge with a JA-30.50 fixed-angle rotor (k-factor: 280) (Beckman Coulter, Brea, CA, USA). The supernatant of the first centrifugation was used for the isolation of the sEVs at 100,000× *g* for 60 min at 4 °C (sEV-pellet). Between each centrifugation the pellets were washed in 1 mL 0.22 µm filtered Dulbecco’s phosphate-buffered saline (DPBS). Samples designated for mass spectrometry were frozen without buffer, while the rest of the samples were resuspended in 100 µL DPBS, thus achieving a 10× concentration from their original volume.

### 2.4. Mass Spectrometry Analysis

Twenty-four samples of lEV and sEV were prepared. Samples were run in technical duplicates. An equal volume of 2× lysis buffer (10% SDS, 100 mM triethylammonium bicarbonate [TEAB], pH 8.5) was added and an appropriate volume of 1× lysis buffer added to 200 µL. Tryptic digestion was performed with the suspension trapping method [22] using S-Trap^TM^ micro spin columns (Protifi, Farmingdale, Huntington, NY, USA), as previously described [16]. Peptide concentration was estimated by tryptophan fluorescence and finally samples were dissolved in 0.1% formic acid at a concentration of 0.25 µg/µL. One µg of each sample (4 µL) was injected as duplicates into a Dionex Ultimate 3000 RSCL nano LC system connected to an Orbitrap Fusion Tribrid mass spectrometer (Thermo Fisher Scientific Instruments, Waltham, MA, USA). The second technical replicate was injected with an interval of a few days from the first injection. Acquisition was performed in MS^1^ with full orbitrap scans at a resolution of 120,000 in the *m*/*z* range 350–1500 and a maximum injection time of 50 ms. Precursor ions with a charge state of 2–7 and an intensity threshold of 1 × 10^4^ were isolated using the quadrupole set with an isolation window of 1.2 *m*/*z* and analyzed in MS^2^ in the linear ion trap with a collision-induced dissociation energy at 35% and a maximum injection time of 70. The MS analysis generated 48 raw files. The raw files were analyzed with MaxQuant version 1.6.5.0 (Max Planck Institute of Biochemistry, Martinsried, Germany: (https://maxquant.net/maxquant/, accessed on 16 January 2026) for label-free quantification (LFQ) analysis [23]. The database searched was Uniprot *Homo sapiens* (filtered and reviewed) (http://www.uniprot.org, accessed on 16 January 2026) downloaded on 17 May 2024. The settings in MaxQuant were with the match between runs function activated, otherwise with the default settings as previously described [24]. The generated protein groups file was entered into Perseus version 1.6.14.0 (Max Planck Institute of Biochemistry, Martinsried, Germany) [25] where data were further filtered for potential contaminants, proteins that were only identified by post-translational modifications and proteins identified in the reverse database. At least two unique peptides were required for protein identification. The LFQ values were Log2 transformed and the combined dataset containing a total of 483 protein identifications were filtered for missing values, giving a set of 84 proteins that were identified in each of the samples analyzed. A summary of the workflow is presented in Supplementary Figure S8. To avoid inflation of CV driven by missing data and prevent imputation artifacts the dataset used for calculation of CV_method_ was restricted to proteins detected in 100% of the samples to accurately measure variation.

CV_dupl_ was calculated using the top 3 most highly expressed proteins.

### 2.5. Nanoparticle Tracking Analysis, NTA

Each sample was measured in 2 replicates with 4 individual measurements in each on the ZetaView NTA system (Particle metrix, Inning am Ammersee, Germany). System settings were validated using 0.1 µm silica beads (Polysciences, Hirschberg, Germany) as per manufacture’s instructions. Optimal particle concentration was determined by pretesting each sample for the ideal particle per frame value (150–250 particles/frame). The following settings were used: Mode: Scatter (488 nm); Scan positions:11 at 60 fps; Camera sensitivity: 80; Shutter: 100; Cell temperature: 23 °C. The video captures were subsequently analyzed with ZetaView Software (8.05.14) with the following settings: Min area: 10; Max area: 1000; Minimum particle brightness: 30.

### 2.6. Western Blot

Western blot was performed to verify CD9+ EV presence in the samples. An EV pool consisting of 12 µL from each sample was used for analysis. The proteins were separated using MiniProtean TGX 4–15% gels (Bio-Rad, Hercules, CA, USA) in Laemmli sample buffer (Bio-Rad, USA) (mixed in a ratio of 1:1) for 5 min at 50 volts and subsequently at 150 volts for 45 min under non-reducing conditions. Following separation, proteins were transferred onto a Bio-Rad 0.45 PVDF Blotting membrane (BioRad, USA) for 60 min at 100 volts and then blocked in 5% (*w*/*v*) skim milk Tris-Glycine buffer. The primary antibody used was a *mouse* monoclonal anti-human CD9 (HYB-SSI 382-01, Statens Serum Institut, Copenhagen S, Denmark), *rabbit* polyclonal anti-AIP1/Alix (Cat. #ABC40) (EMD, Millipore, Darmstadt, Germany), and a *goat* anti-human APO-B 48/100 (#K45253G, Meridian Life Science, Cincinnati, OH, USA), diluted 1:500 in 5% skim milk Tris-Glycine buffer. The membrane was incubated with this antibody solution overnight at 4 °C. Secondary labeling was carried out using a horseradish peroxidase-conjugated *goat* anti-mouse antibody (1:30,000, Dako, Glostrup, Denmark), *goat* anti-rabbit antibody (1:30,000 Cat. #170-5046) (Biorad, Hercules, CA, USA) and *rabbit* anti-goat (1:10,000, #81-1620, Invitrogen, Waltham, MA, USA) for 2 h at room temperature. For the development and detection of the proteins, Lumi-Light Plus Western Blotting substrate detection reagent (Roche, Basel, Switzerland) was used, and the detection process was conducted using a PXi 4 system (Syngene, Cambridge, UK) equipped with GeneSys software version 1.5.4.0 (Syngene, UK).

### 2.7. Transmission Electron Microscopy, TEM

TEM was used to assess the detailed morphology and structure of the EVs with immuno-gold labeling against CD9, as previously described [26]. In short, 5 μL of EV isolate was mounted on a grid (SPI Supplies, West Chester, PA, USA) and stained with one drop of 1% (*w*/*v*) phosphotungstic acid (pH 7.0, Ted Pella, Caspilor AB, Vendelsö, Sweden), and subsequently blotted dry on filter paper. To visualize the surface presence of CD9 on EVs, Immunoelectron microscopy, IEM, was performed. The vesicles were transferred to a grid as described above and blocked with ovalbumin. The grids were then incubated with primary antibody for CD9 1:50 (HYB-SSI 382-01, Statens Serum Institut, Denmark). Hereafter, the grids were incubated with secondary goat anti mouse antibodies conjugated with 10 nm colloidal gold (1:25, British BioCell, Cardiff, UK) incubated 3 × 10 min at room temperature in three drops of 1% cold fish gelatin (Sigma-Aldrich, St. Louis, MO, USA) in PBS, washed on three drops PBS, stained with 1% phosphotungstic acid, pH7.0 and blotted dry. The grids were then stained with 1% (*w*/*v*) phosphotungstic acid at pH 7.0 and blotted dry. Images were obtained with a transmission electron microscope (JEOL 1400, Tokyo, Japan) operated at 60 keV and equipped with a TVIPS TemCam FX416 digital camera (TVIPS, Gauting, Germany).

### 2.8. EV-Array

The EV-Array analysis was carried out on plasma samples as described earlier [8,27]. In brief, printed slides were first blocked with 50 mM ethanolamine, 100 mM Tris, and 0.1% SDS (pH 9.0). Ten microliters of plasma or serum, diluted 1:10 in wash buffer (0.05% Tween 20 in PBS) were then applied and incubated in Multi-Well Hybridization Cassettes (ArrayIt Corporation, Sunnyvale, CA, USA) at room temperature for 2 h on an orbital shaker (450 rpm), followed by an overnight incubation at 4 °C without agitation. After rinsing, the slides were incubated for 2 h with a cocktail of biotinylated anti-human CD9, CD63, and CD81 antibodies (LifeSpan BioSciences, Seattle, WA, USA), diluted 1:1500 in wash buffer. After another wash step, a 30 min incubation with Cy5-labeled streptavidin (Life Technologies, Carlsbad, CA, USA), also at 1:1500 dilution, was used for signal development. Finally, slides were washed one last time, rinsed with deionized water, and dried using a Microarray High-Speed Centrifuge (ArrayIt Corporation, CA, USA). Scanning at 635 nm and subsequent data acquisition were performed as described in the original protocol. To facilitate the phenotyping of EVs, a total of 17 antibodies were selected: CD9 (Ancell, Bayport, MN, USA, Cat.# 156-020, Clone: SN4/C3-3A2), CD4 (R&D Systems, Minneapolis, MN, USA, Cat.# MAB379, Clone: 34930), CD63 (Biorad, Hercules, CA, USA, Cat.# MCA2142, Clone: MEM-259), CD8a (R&D Systems, Cat.# MAB1509, Clone: 37006), CD81 (Ancell, Cat.# 302-020, Clone: 1.3.3.22), HLA ABC (Biolegend, San Diego, CA, USA, Cat.# 311402, Clone: W6/32), CD142 (R&D Systems, Cat#. MAB2339, Clone: 323514), Flotillin-1 (Abcam, Cambridge, UK, Cat.# ab41927) CD42a (LS Bio, Seattle, WA, USA, Cat.# LS-C45240), CD45 (R&D Systems Cat.# MAB1430, Clone: 2D1), CD106 (R&D Systems Cat.# MAB809, Clone:HAE-2Z), MIC A/B (R&D Systems Cat.# MAB13001 Clone: 159207), ICAM-1 (eBioscience, San Diego, CA, USA, Cat.# BMS1011, Clone: R6.5), HLA DR/DP/DQ (Capricobio, Norcross, GA, USA, Cat.# 104701 Clone: HB-145/IVA12), CD31 (R&D Systems, Cat.# AF806, Polyclonal, TNF RII (R&D Systems, Cat.# MAB726 Clone: 22210) and TNF RI (R&D Systems, Cat.# MAB225, Clone:16803). The mean signal intensities from each antibody’s triplicate spots were background-corrected by subtracting the average signal from blank wells (no sample). For each capture antibody, these corrected values were then normalized against the average signal of negative control spots (PBS only). Prior to plotting and statistical testing, antibody signals were log_2_-transformed.

### 2.9. Statistics

Data were analyzed with GraphPad Prism 10 (GraphPad Software, La Jolla, CA, USA). All diagrams are presented either as mean ± SD or as representative individual experiments. A *p*-value > 0.05 was considered not significant (ns); *p* <0.05 *, *p* <0.01 **, *p* <0.001 *** and *p* <0.0001 ****. MS data were log_2_-transformed prior to statistical testing. All data were tested for normality and non-parametric tests were used where appropriate. Paired Student’s *t*-tests (or Wilcoxon signed-rank tests where appropriate) were used to compare AM vs. PM samples; unpaired *t*-tests or Mann–Whitney *U*-tests were used for comparisons between lEVs and sEVs with FDR correction for multiple testing where applicable. PCA plots were created using SIMCA 17 (Sartorius Stedim, Göttingen, Germany). The coefficient of variation (CV) was defined as CV = 100 × (SD/mean) [12]. A pooled CV was calculated as the square root of the mean of the squared CVs (RMS) for all CVs, except for CV_method_ and CV_biological_ for MS and EV-Array where median CV was used. We estimated duplicate precision (CV_dupl_) as the coefficient of variation by taking the square root of the average of the squared relative differences between paired measurements (each difference ×1 minus ×2 divided by the pair mean), divided by two. For each method, CV_method_ quantified repeated measurements of the same prepared sample (instrument/assay repeatability; CV_dupl_ captured variability introduced by duplicate blood tubes and independent downstream processing collected at the same time point (pre-analytical + isolation/handling + measurement); CV_am vs. pm_ captured variability between paired morning vs. afternoon collections, i.e., the variation in two samples collected at two different time points. CV_biological_ reflected between-subject variability.

## 3. Results

All included individuals completed the blood sampling. For each sample two duplicates were centrifuged to isolate sEVs and lEVs as described in Methods. Each of these samples were investigated by different methods a number of times (dependent on the methodology) to estimate the CV_method_ and from the two duplicates the CV for two samples was calculated (CV_dupl_). Finally, the difference between the samples from morning (AM) and afternoon (PM) was compared and CV_am vs. pm_ calculated. For comparison, the biological differences, i.e., between-subject variation, (CV_biological_) were estimated from the six individuals. In the following section, these variations for the different measurements are first described, and these results are summarized at the end.

### 3.1. Characterization of Extracellular Vesicles

**Figure 2:**
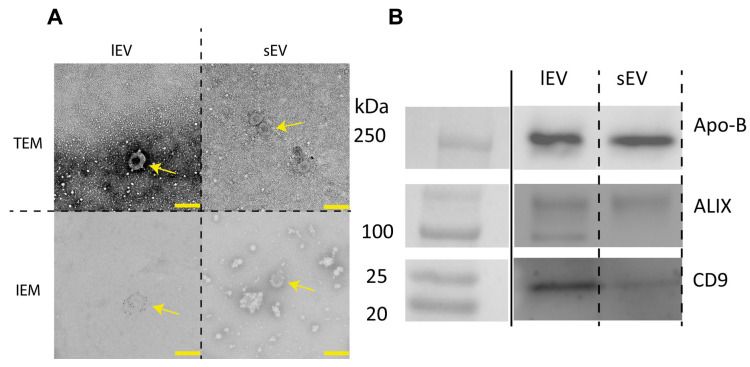
Morphologic and protein characterization of EV samples. (**A**) TEM and IEM images of isolated EV preparations. TEM showing vesicular structures around 200 nm. IEM images showing CD9+ extracellular vesicles with marker antibodies connected to the outer membrane with some debris present. Yellow bar: 200 nm. All images acquired at 20,000×, Panels displayed at the same magnification. Yellow arrows indicate objects of interest. (**B**) Western blot of pooled participant samples confirming the presence of CD9 and ALIX and lipoprotein marker Apo-B.

To assess both sample purity and the presence of EVs, both TEM and IEM were conducted. The TEM images showed heterogeneous membrane-bound structures in both lEV and sEV preparations, with particle sizes predominantly in the 100–200 nm range (Figure 2A). IEM labeling with anti-CD9 demonstrated CD9-positive membrane structures consistent with EVs in both fractions. Western blots of pooled samples confirmed the presence of the EV-associated markers CD9 and ALIX, together with the lipoprotein marker ApoB, indicating co-isolation of EVs and lipoproteins (Figure 2A; Supplementary Figures S1–S4).

### 3.2. Particle Counting

Each of the initial duplicates was examined by NTA, analyzing each sample 4 times. For Particle size, the CV_method_ was calculated to 3.3% while CV_dupl_ 5.1%, CV_biological_ 11.5%, and CV_am vs. pm_ was 8.8%. However, for particle concentration the CV_method_ was 7.9%, CV_dupl_ 47.0%, CV_biological_ 77.1% CV_am vs. pm_ 70.5% showing a higher degree of variability both methodologically and across individuals in particle concentration.

**Figure 3:**
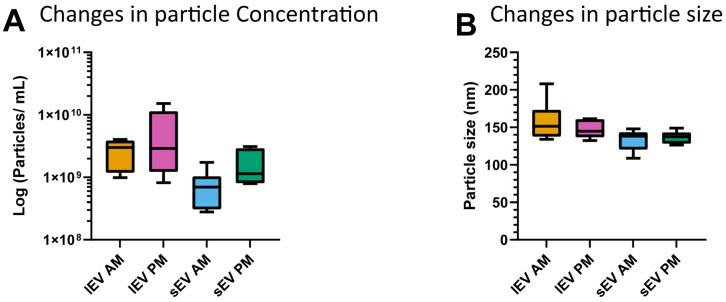
Characteristics of variation in isolated particles. Changes in particle concentration and particle size between AM and PM (**A**,**B**).

Morning and Afternoon Variation. Fluctuations in particle concentration during morning and afternoon periods were also measured with the NTA system. Mean particle size and concentration values are summarized in Supplementary Table S1. Analysis of cumulative NTA data indicated no systematic changes in either particle concentration nor size between AM and PM for both the lEV and sEV preparations (Figure 3). Dot plots for individual participant variation can be found in the Supplementary Materials (Figure S5).

### 3.3. EV Surface Marker Variation

The EV-Array is performed on plasma without isolation of EVs, and each of the samples were, therefore, measured in two sets of triplicates by the EV-Array on different plates analyzing each sample six times in total. The mean CV_method_ was calculated to be 22.6%, while the average CV_dupl_ was 21.1%, the mean CV_biological_ was calculated to >100%, and lastly the CV_am vs. pm_ 21.7%.

**Figure 4:**
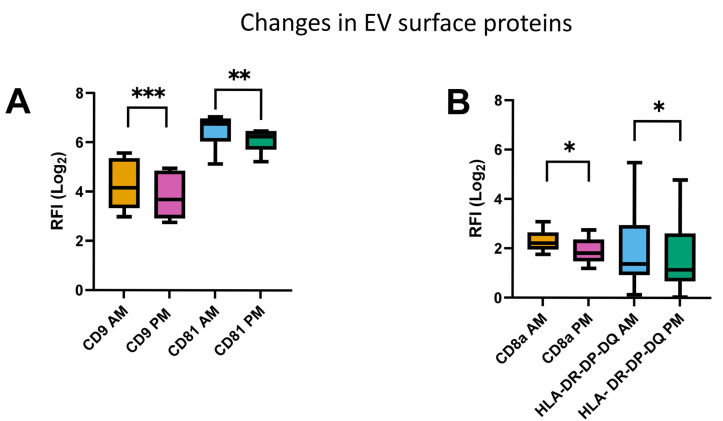
Characteristics of significant changes in extracellular vesicle surface proteins between AM and PM (**A**,**B**). * *p* < 0.05, ** *p* < 0.01, *** *p* < 0.001.

Morning and Afternoon Variation. The tetraspanins CD9, CD63 and CD81 were chosen according to the MISEV guidelines to identify EVs with the remaining markers used as generic EV associated proteins. Very small but significant changes were found for CD9 and CD81 indicating alterations in CD9^+^ and CD81^+^ vesicle release between AM and PM (Figure 4A,B). Although CD9 and CD81 showed statistical significance, the absolute effect sizes were small (<0.5 log_2_ RFI) and were within the magnitude of CV_dupl_, suggesting limited impact. Detailed numerical values for CD9, CD81, CD8a and HLA-DR/DP/DQ are provided in Supplementary Table S2. A heatmap of all the chosen surface proteins and their dot plots can be found in the Supplementary Materials (Supplementary Figures S6 and S7).

### 3.4. EV Cargo Variation

For mass spectrometry each sample was aliquoted into two and run in technical duplicates. Using mass spectrometry, we identified a total of 270 different proteins from our EV samples. For the calculations of CV, only 100% valid values were used for proteins detected in the participants, 84 of which were identified. Each sample was analyzed in technical duplicates. The median CV_method_ was calculated to 8.2% while CV_dupl_ was 20.1%, the median CV_biological_ was calculated to 34.7% and CV_am vs. pm_ was 14.01%. The top 10 differentially expressed proteins in the lEV and sEV groups and associated volcano plot can be seen in Supplementary Table S3 and Supplementary Figure S9.

**Figure 5:**
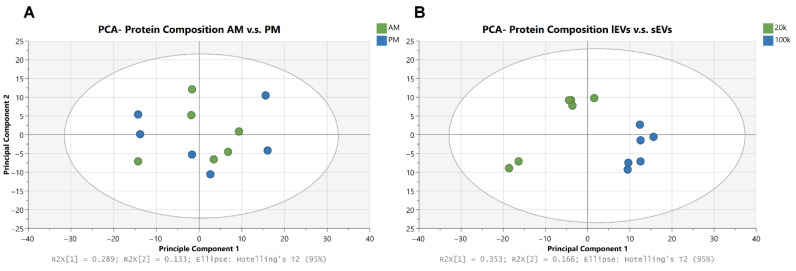
PCA plot of proteome alterations of all isolated EVs between AM and PM (**A**) and alteration between the cargo of sEVs and lEVs (**B**).

Morning and Afternoon Variation. Principal component analysis (PCA) of the combined EV populations collected AM and PM showed a lack of clustering and group separation (Figure 5A). Similarly, statistical analysis showed a lack of proteins with a significant q-value. No protein cargo variations were observed when comparing sEV or lEV collected at AM and PM to each other (Supplementary Figure S10). A subsequent analysis of the differences in the cargo between sEVs and lEVs using PCA (Figure 5B) showed clear signs of separation between lEVs and sEVs; statistical analysis further confirms the difference in proteomes.

### 3.5. Overview of Variations

The determination of particle size had a rather low variation at all steps whereas determination of concentrations has a high variation, especially between samples (CV_dupl_ and CV_am vs. pm_) (Table 1). Mass spectrometry has a lower variation between samples, and CV_dupl_ and CV_am vs. pm_ are quite similar. For EV-Array, CV_method_ and CV_dupl_ are of the same size because it does not include an isolation step, and CV_am vs. pm_ is also similar even though some of the markers revealed a small but significant difference. For all measurements, the biological variation was higher or much higher than the variation between samples from the same person.

## 4. Discussion

This study aimed to explore the variation in various assays frequently used in the quest for new EV-derived biomarkers. The analysis of variation encompassed (i) the method (CV_method_), which assesses variation between determinations within the same sample; (ii) two distinct samples (CV_dupl_) that involve the isolation of EVs (except the EV-Array for surface markers); (iii) variations between two samples collected at two different time points, i.e., in the morning and afternoon (CV_am vs. pm_); and, ultimately, (iv) differences between subjects (CV_biological_), respectively. In a clinical setting focused on identifying new biomarkers, understanding the distinctions between two samples is crucial for assessing the potential of a biomarker to differentiate between cases and controls. This is relevant considering that patients may arrive for blood sampling in the morning or in the afternoon.

We assessed nanoparticle tracking analysis (NTA), a multiplex EV protein microarray (EV-Array), and label-free mass spectrometry (MS) for EV cargo. The measurements displayed a low to acceptable degree of variation (CV_method_) for the MS and particle size measurements even down to single digits, but a higher variation for the EV-Array, in line with other studies [28,29]. Even though certain levels of variation are slightly higher than reported in other studies, these can be attributed to different analytical approaches such as using TMT labeling in case of MS. The variation for the EV-Array was also within generally acceptable limits of less than 25% variation [7,17,28,30]. For CV_dupl_ including the isolation of EVs the variation increased. One of the highest degrees of variation was observed for the CV_dupl_ for the NTA analysis. As NTA does not currently allow for discrimination between EVs and other contaminating particles, and the removal of lipoproteins and EV labeling were suboptimal (Figure 3), some of the variation in particle concentration could be reasonably explained by co-isolated contaminants such as lipoproteins [31]. Our investigation identified lipoprotein contamination during extracellular vesicle isolation (Figure 2), while adhering to the identical techniques established by Nielsen and coworkers [26]. ApoB co-isolation is a recognized limitation of differential ultracentrifugation in plasma [32,33]. Future work will evaluate alternative purification techniques (SEC and/or density gradients) to reduce lipoprotein presence where subtype-specific biological inference is the primary objective. However, stringent purification protocols are often associated with loss of EVs and thus signal strength.

The variation in size determination exhibited minimal decrease suggesting that the isolation stage may lose particles, but not in a size-selective manner. In our MS analyses, CV_dupl_ did not rise significantly compared to concentration determination for NTA, suggesting that variations in lipoproteins with low protein content had a reduced impact on the assessment of EV cargo. For the EV-Array, CV_method_ and CV_dupl_ were similar in size due to the measurements being conducted on plasma, as no isolation of EVs was needed. The primary source of variation among all approaches was the CV_biological_, which has been thoroughly documented in the literature. [7,13,15,31,34], showing that although the analytical variation is far from negligible, it is considerably lower than CV_biological_. No studies employed characterized (CV_dupl_), rendering direct comparisons difficult. Our findings are overall consistent with previous reports that biological variability in EVs exceeds technical variability. Newman et al. quantified within- and between-subject variability in serum-derived sEV concentration and cargo in healthy volunteers and reported modest but significant diurnal variation for selected markers [21]. Oeyen et al. assessed technical and biological variation in urinary EV proteomics further supporting that biological CV exceeds analytical CV [20].

We observed negligible discrepancies between AM and PM sample collections, akin to the variances observed between two samples (CV_dupl_.). No noticeable trends in particle size or concentration were seen in NTA. Bazié and colleagues observed a notable 40 nm reduction in lEV size and a decline in sEV concentration from morning to evening; however, these alterations were not statistically significant for sEV size or lEV concentration [14]. Variables such as sample collection intervals may affect these discrepancies, particularly when employing flow cytometry with antibody-labeled extracellular vesicles [35].

Mass spectrometry revealed no substantial alterations in EV cargo between morning and afternoon samples, in contrast to prior research that observed diurnal variations in plasma proteins and EVs [21,36,37,38]. For example, Yeung et al. identified EV protein rhythms through 24 h synchronized cell cultures, and Specht et al. observed circadian plasma proteins using hourly sampling over 24 h [36,39]. Additionally, Danielson et al. used nanoscale flow cytometry to show shifts in the relative size and abundance of plasma EV populations across three time points between morning and evening in healthy adults, while Newman et al. observed diurnal variation in global sEV markers (CD81) and liver-specific EVs (ASGR1) between morning and afternoon samples [21,38]. We did not detect systematic diurnal changes in NTA particle counts, size, or cargo proteins within a narrower time window (08:00–13:00). These differences could be a combination of factors such as shorter sampling interval in our study, the use of differential ultracentrifugation to separate lEV and sEV fractions, and methodological differences. Our data therefore do not necessarily contradict prior evidence for circadian influences on EVs, but rather suggest that within standard daytime blood collection hours, AM to PM variation is modest compared to between-subject variability.

Our results also confirmed a difference between the proteome of lEVs and sEVs. This has been described extensively in the literature and will not be discussed [40]. Although the mean variation in all the investigated surface markers measured by EV-Array did not indicate a systematic difference between the two measured time points, a diurnal modification of a few EV surface proteins was observed, characterized by decreased CD9+ and CD81+ markers, indicating a slight reduction in EVs based on the time of day. The EV-Array findings also indicated a slight decrease in CD8a+ and HLA DP/DQ/DR levels partially in line with other studies [41]. Despite the identification of a statistical significance for daytime variations for these few biomarkers, the absolute changes were negligible and improbable to possess therapeutic or biological importance as biomarker studies typically target a >1 Log_2_ in protein expression for potential biomarkers [42,43].

The main limitation of this study is the small sample size (n = 6), which reduces the ability to detect smaller changes and can lead to imprecise estimates of CV_biological_ and CV_am vs. pm_. Differential centrifugation is insufficient for the complete separation of sEV and lEV populations, which may lead to an inflated CV_biological_ due to the presence of mixed subpopulations. The study collected data at only two time points on the same day, restricting a thorough longitudinal analysis of diurnal vesicle variation; still, our research aimed to demonstrate potential differences between patients seen in the morning and those seen in the afternoon. Using proteins only detected in 100% of the samples will under-represent low abundance proteins. However, for variation studies the use of proteins detected in 100% of the samples is necessary [44].

## 5. Conclusions

This exploratory pilot study presents results of a thorough effort to measure methodological and biological variance. For protein cargo, no discernible differences between morning and afternoon times were found, despite individual variances in particle size and concentration, thus indicating a degree of stability of EV cargo in the specific timeframe. However, minor, statistically significant, but clinically insignificant, changes in a few biomarkers, i.e., CD9+, CD81+, CD8a+ and HLA DP/DQ/DR+ vesicles were detected by EV-Array. Variation between samples in the same persons for EV cargo and surface marker displayed a reasonably low variation. Despite this, due to the small sample size in this study, small effects of daytime EV fluctuations cannot be excluded.

## Figures and Tables

**Table 1:** An overview of the calculated Coefficients of variation for each method applied in the study.

Coefficients of Variation Overview					
Method	Measurement	CV_method_	CV_dupl_	CV_biological_	CV_am vs. pm_
Zetaview NTA	Particle Size (nm)	3.3%	5.1%	11.5%	8.8%
Zetaview NTA	Particle Concentration	7.9%	47.0%	77.1%	70.5%
EV-Array	RFI	22.6%	21.1%	>100%	21.7%
Mass spectrometry	LFQ	8.2%	20.1%	34.7%	14.0%

## Data Availability

The original contributions presented in this study are included in the article/Supplementary Material. Further inquiries can be directed to the corresponding author.

## Supplementary Materials

The following supporting information can be downloaded at: https://www.mdpi.com/article/10.3390/cimb48010120/s1.

## Abbreviations

The following abbreviations are used in this manuscript:

ALIX	Apoptosis-linked gene 2-interacting protein X
AM	Morning (Ante Meridiem)
APO-B	Apolipoprotein B
BMI	Body Mass Index
CD	Cluster of Differentiation
CI	Confidence Interval
CV	Coefficient of Variation
CV_am vs. pm_	Coefficient of variation between morning and afternoon samples
CV_biological_	Biological coefficient of variation (between subjects)
CV_dupl_	Coefficient of variation between duplicate samples
CV_method_	Coefficient of variation within method (technical variation)
DPBS	Dulbecco’s Phosphate-Buffered Saline
EV	Extracellular Vesicle
FDR	False Discovery Rate
HLA	Human Leukocyte Antigen
IEM	Immunoelectron Microscopy
ICAM-1	Intercellular Adhesion Molecule-1
IQR	Interquartile Range
LC-MS/MS	Liquid Chromatography–Tandem Mass Spectrometry
LFQ	Label-Free Quantification
lEV	Large Extracellular Vesicle
MISEV	Minimal Information for Studies of Extracellular Vesicles
MS	Mass Spectrometry
NTA	Nanoparticle Tracking Analysis
PBS	Phosphate-Buffered Saline
PCA	Principal Component Analysis
PM	Afternoon (Post Meridiem)
PPP	Platelet-Poor Plasma
PVDF	Polyvinylidene Difluoride
RFI	Relative Fluorescence Intensity
SD	Standard Deviation
SDS	Sodium Dodecyl Sulfate
sEV	Small Extracellular Vesicle
TEAB	Triethylammonium Bicarbonate
TEM	Transmission Electron Microscopy
TNF RI/RII	Tumor Necrosis Factor Receptor I/II
Tris	Tris(hydroxymethyl)aminomethane
TMT	Tandem Mass Tag
