# Supplementary material for: Methodological and Short-Term Diurnal Variation in Surface and Cargo Proteins in Plasma Extracellular Vesicles
Source: Curr Issues Mol Biol. 2026 Jan 22;48(1):120. doi: 10.3390/cimb48010120 (PMC12840092; doi:10.3390/cimb48010120)
Supplement: Supplementary file 1 [file cimb-48-00120-s001.zip › Supplementary information- CIMBv4.pdf]

Supplementary information

Supplementary figure S1 2
Supplementary figure S2 3
Supplementary figure S3 4
Supplementary figure S4 5
Supplementary figure S5 6
Supplementary figure S6 6
Supplementary figure S7 7
Supplementary figure S8 8
Supplementary figure S9 9
Supplementary figure S10 10
Supplementary table S1 11
Supplementary table S2 12
Supplementary table S3 13

Supplementary figure S1

Raw IEM/TEM images (figure 2a).

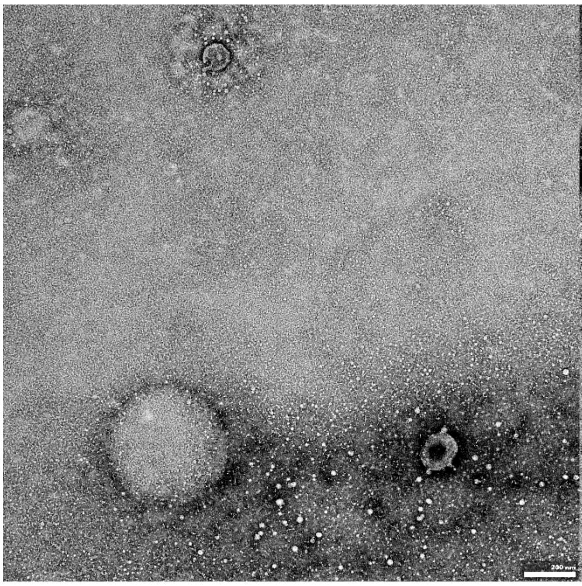

Figure 2.a 1

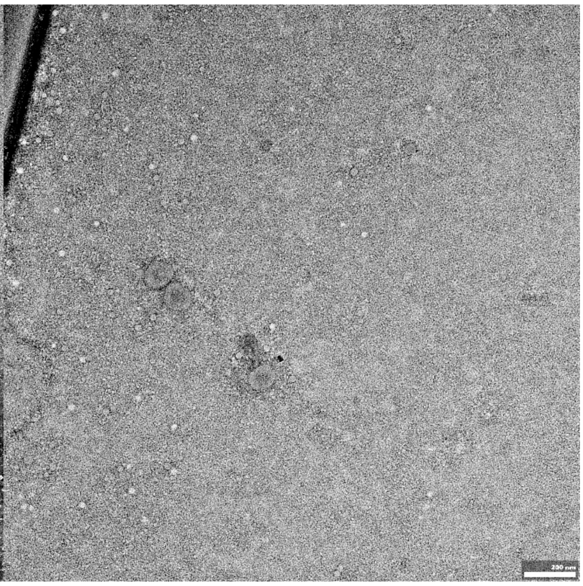

Figure 2.a 2

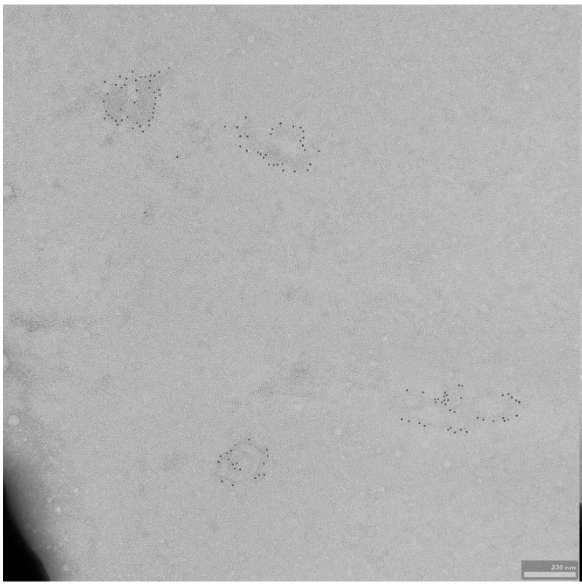

Figure 2.a 3

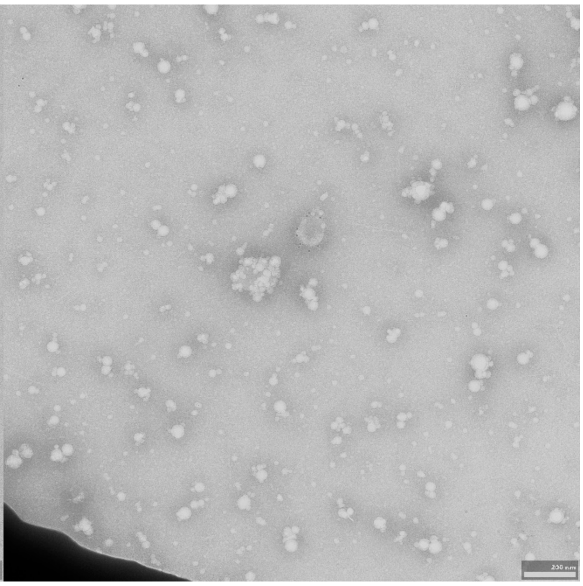

Figure 2.a 4

Supplementary figure S2

Raw Western blot images of ApoB.

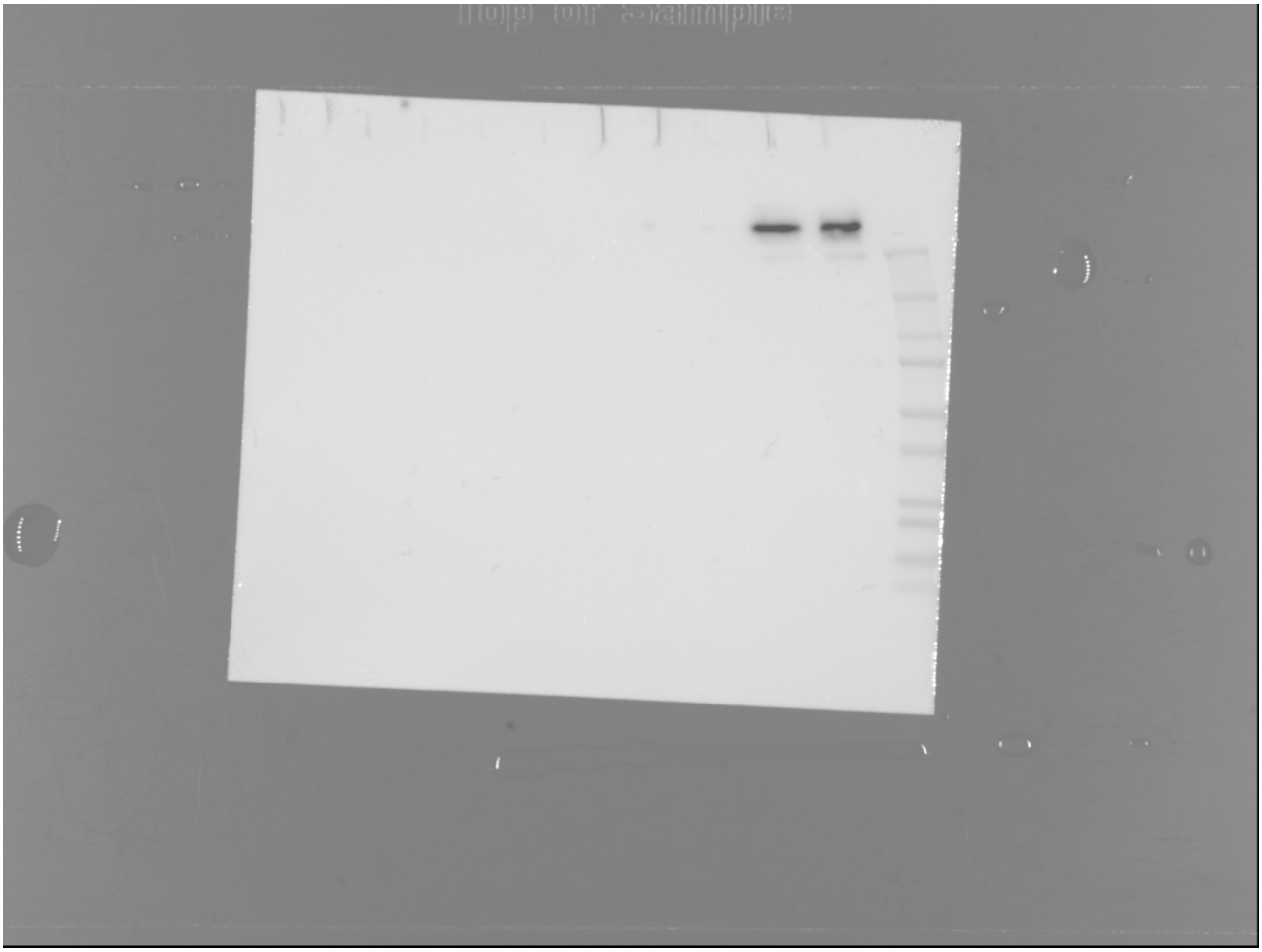

Supplementary figure S3

Raw western blot image of CD9.

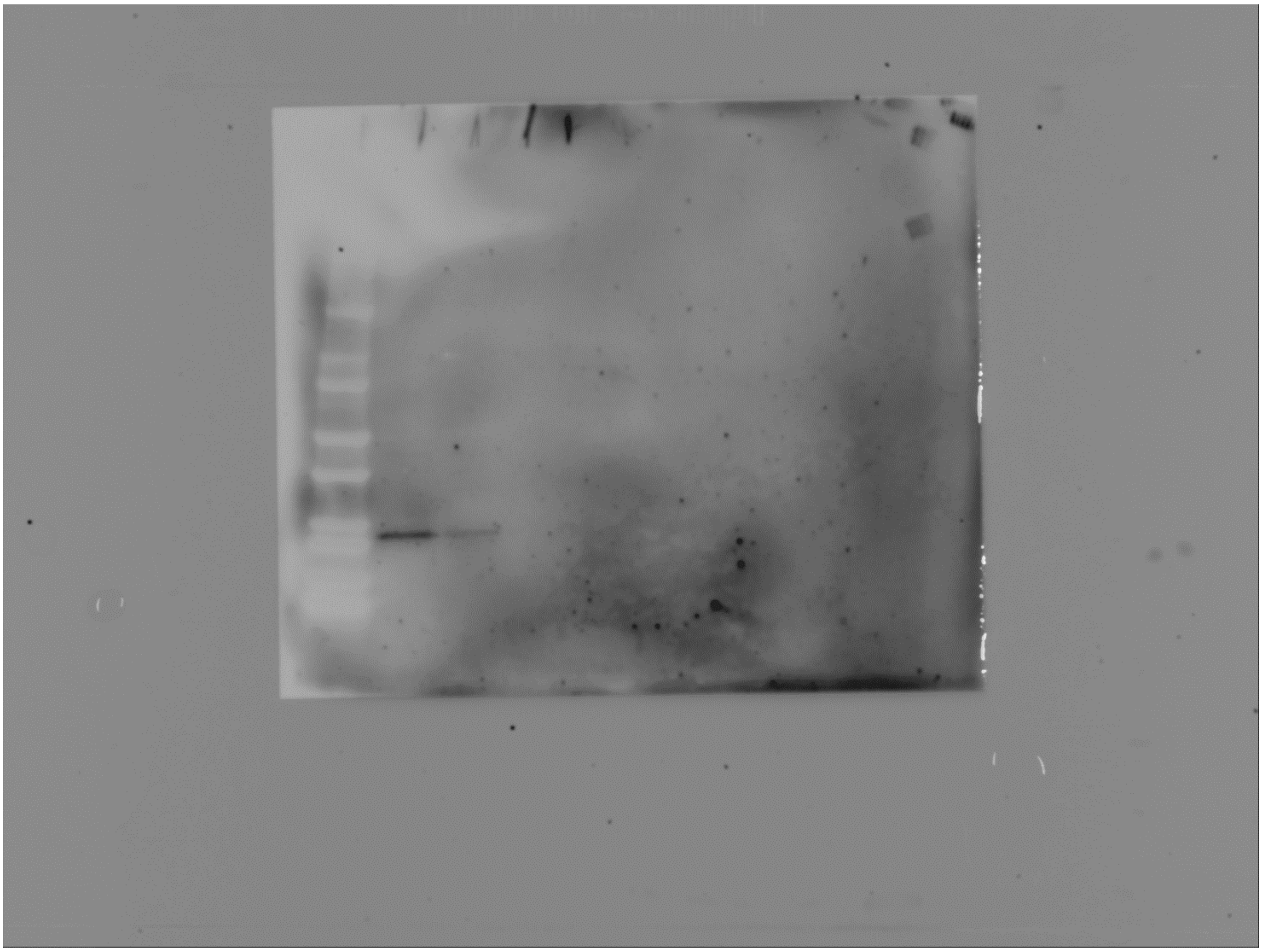

Supplementary figure S4

Raw western blot image of ALIX.

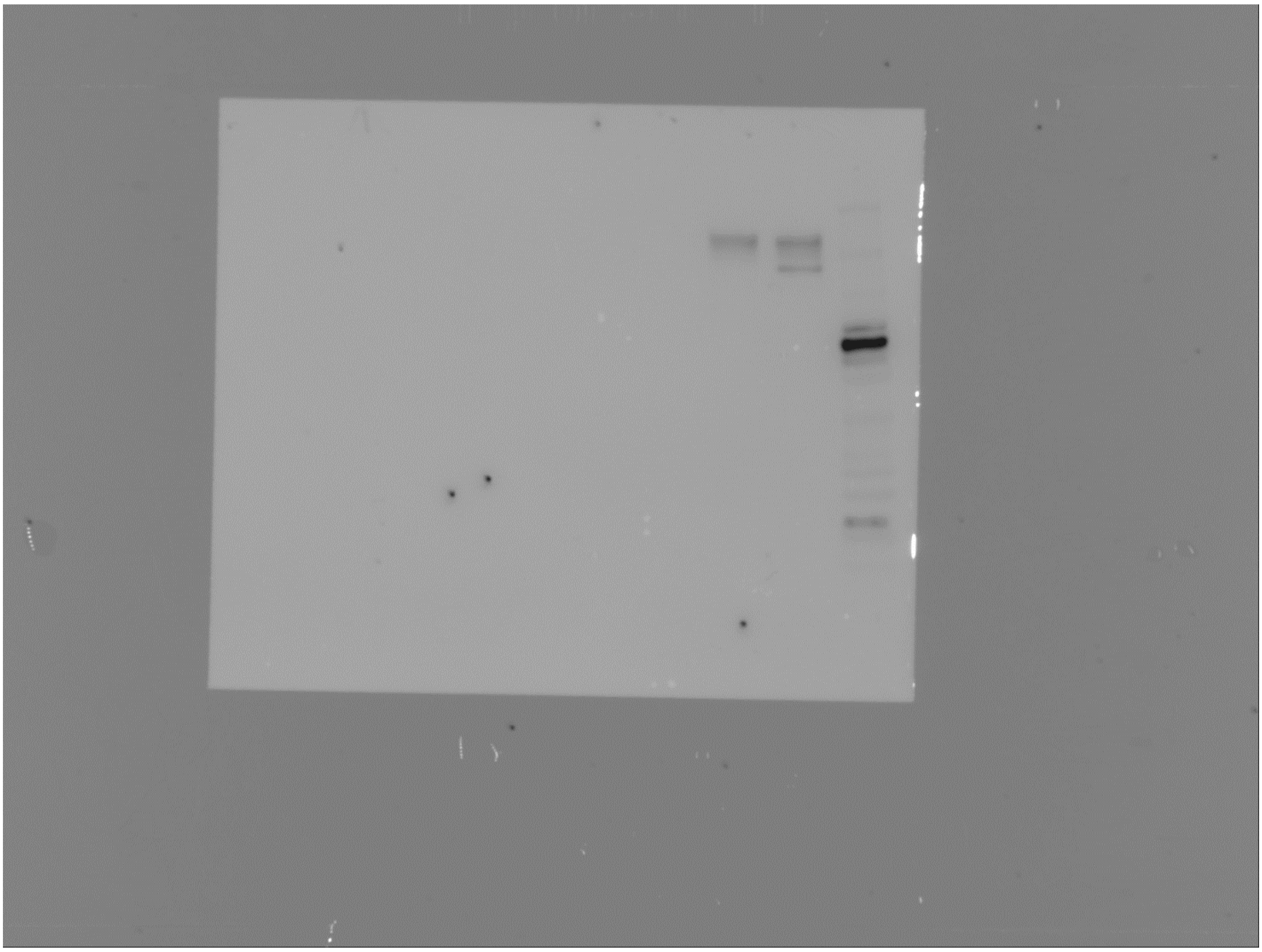

Supplementary figure S5

Dotplot of individual participant particle variation throughout the day.

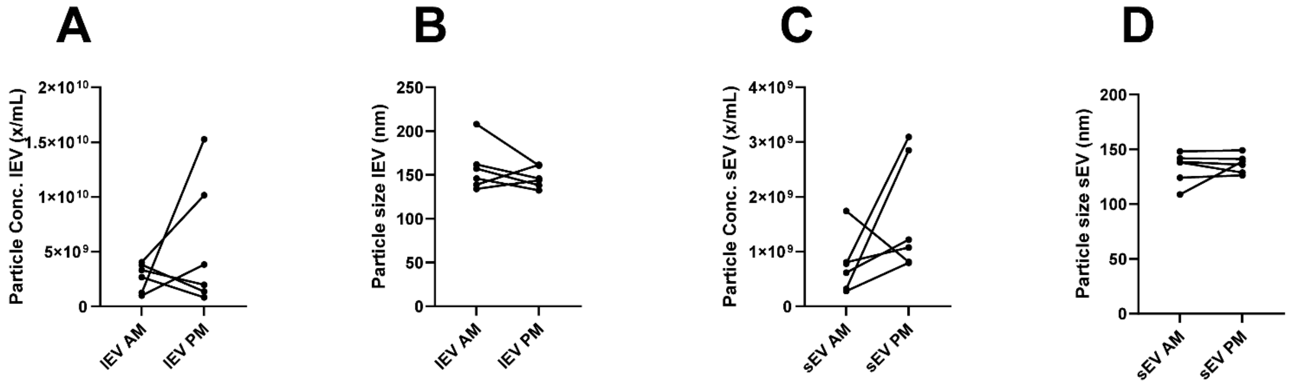

Supplementary figure S6

Heatmap of the tested surface EV associated proteins.

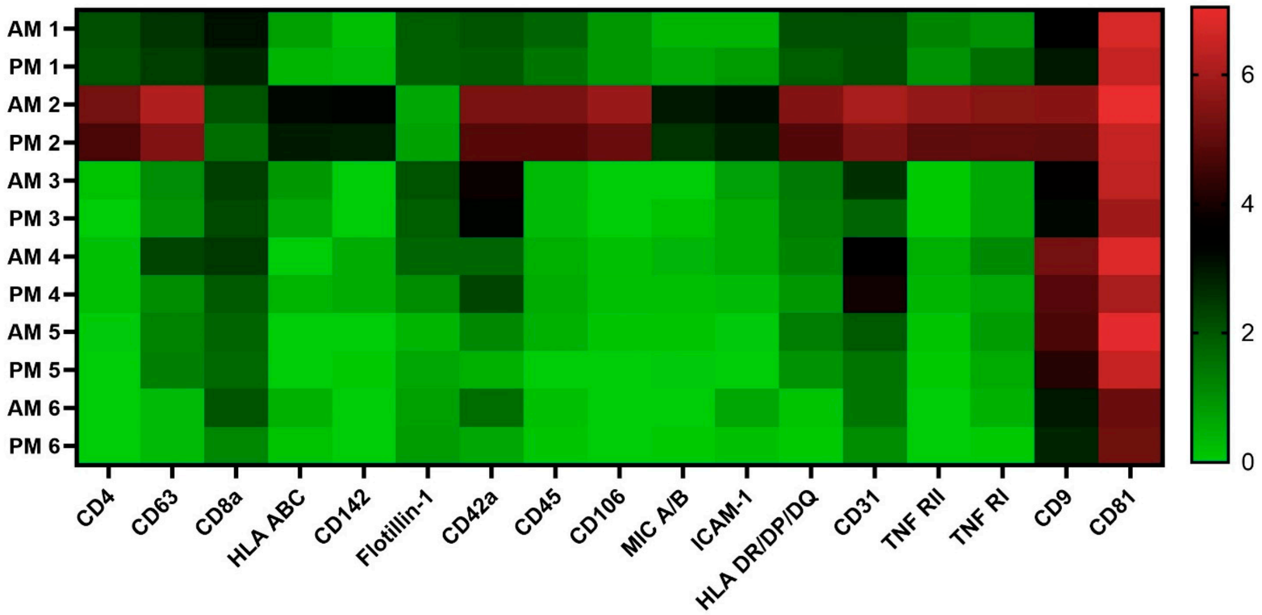

Supplementary figure S7

Estimation plots of the tested surface EV associated proteins.

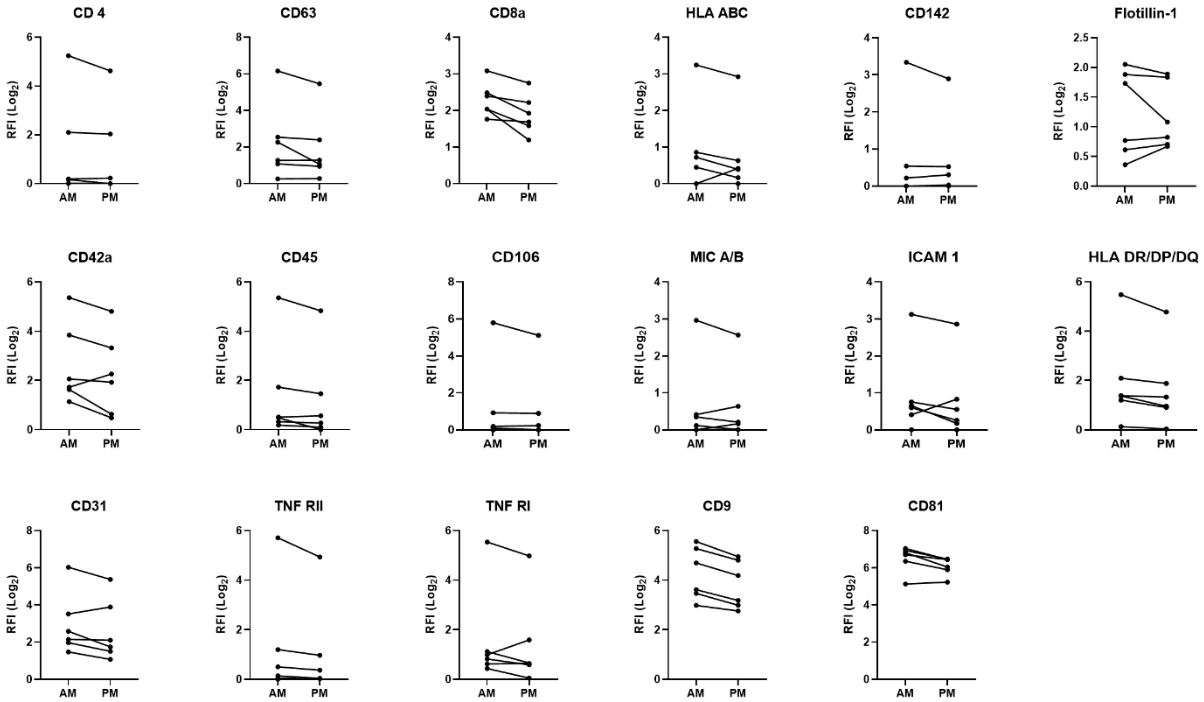

Supplementary figure S8
Workflow in Perseus.

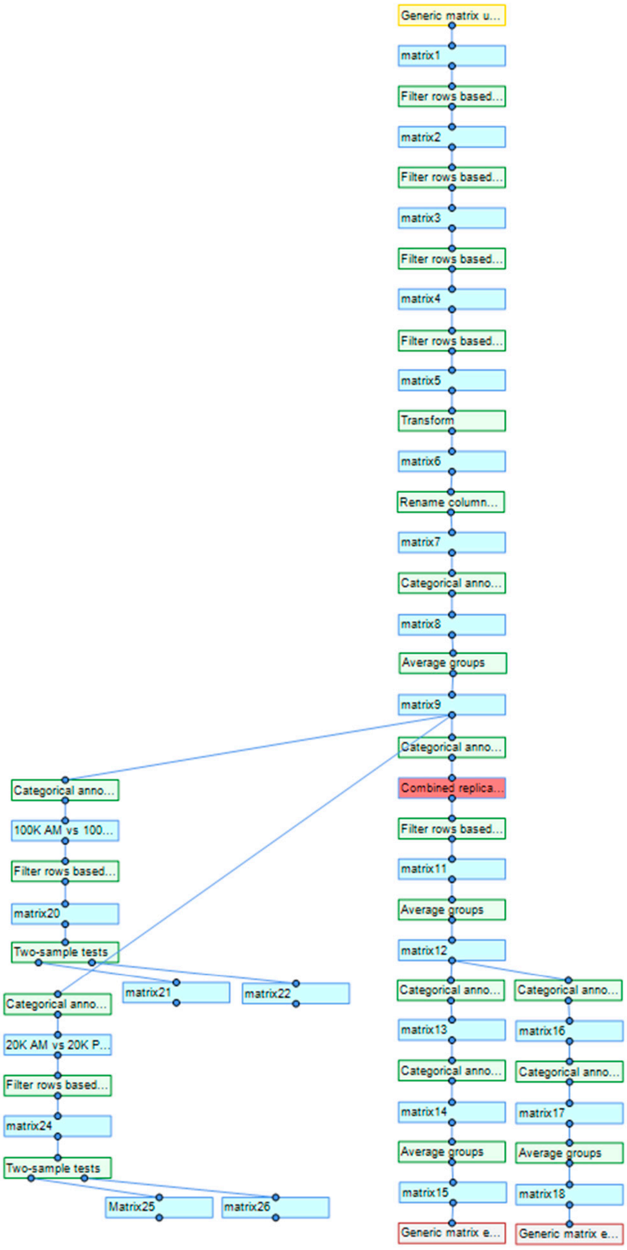

Supplementary figure S9

Volcano plot of the differences in the lEV and sEV groups.

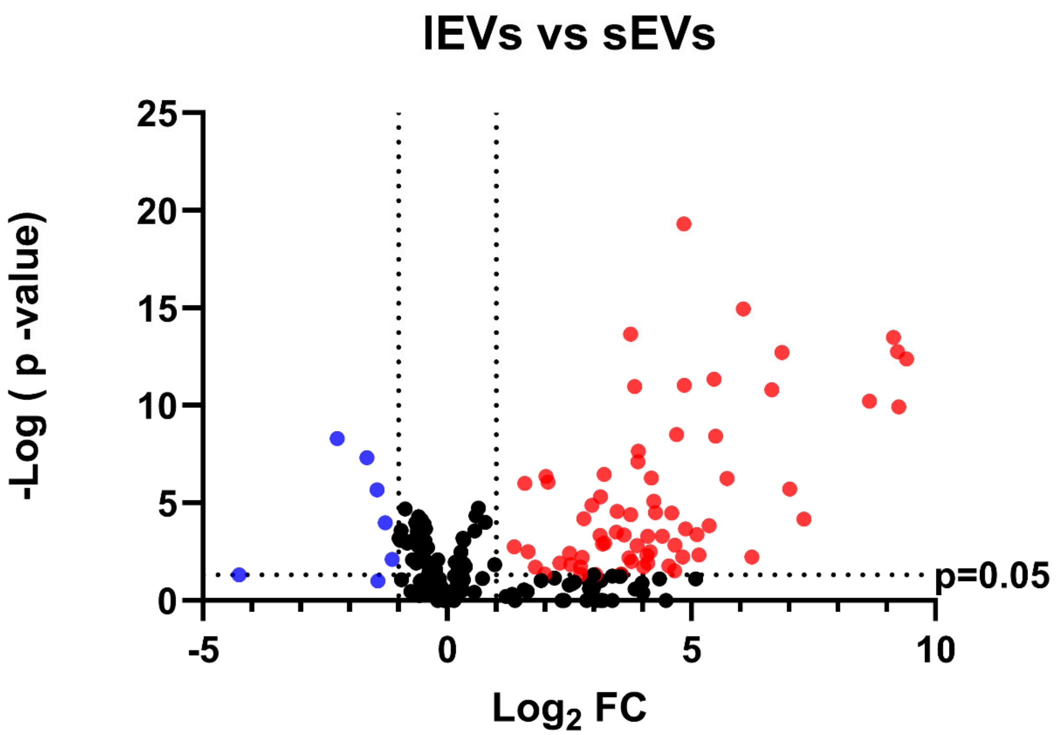

Supplementary figure S10

Volcano plot of the differences in the AM and PM groups.

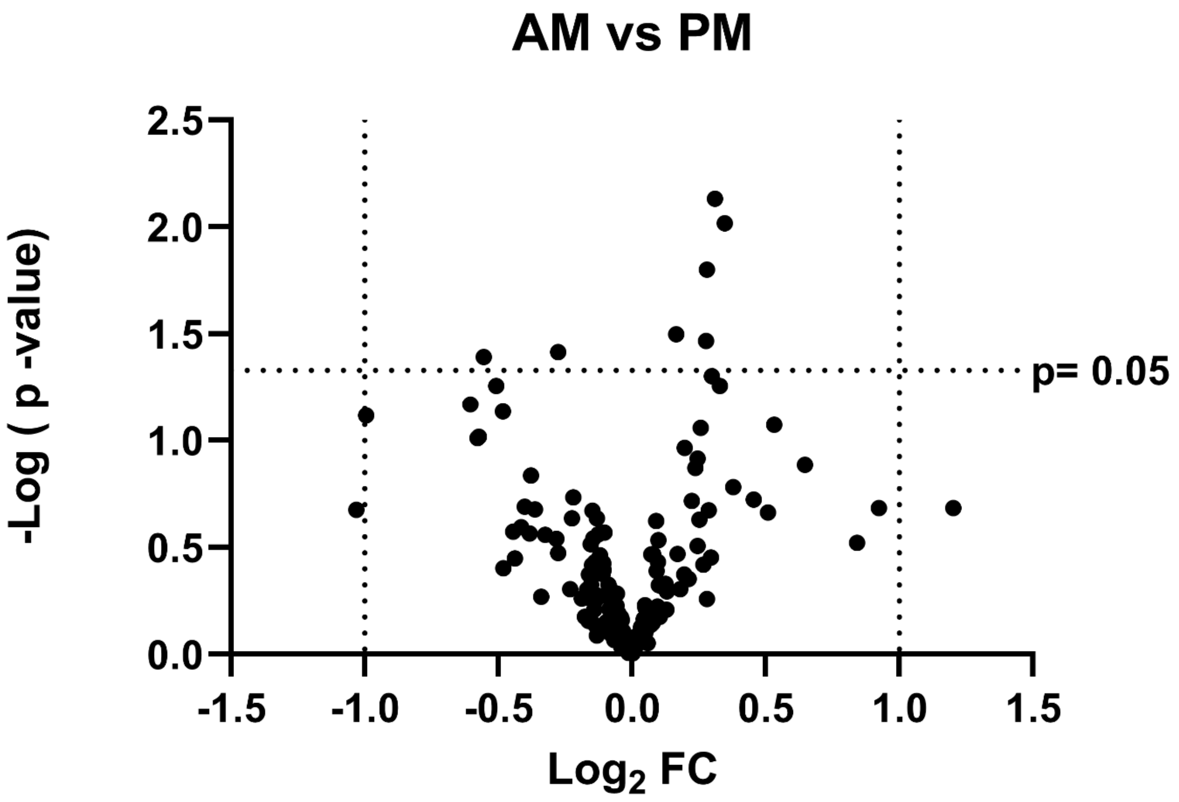

Supplementary table S1

Variations in particle size and concentration in the large and small EV groups at 08:00 (AM) and 13:00 (PM)

**Changes in plasma nanoparticles**								
	**Particle count (Particles/mL)**				**Mean Size (nm)**			
	**AM**	**PM**	**Difference**	***p***	**AM**	**PM**	**Difference**	***p***
lEV	$2.68x10^9$ ($1.3x10^9$)	$5.56x10^9$ ($5.8x10^9$)	ns	0.318	157.7 (26.8)	147.7 (11.8)	ns	0.335
sEV	$7.56x10^8$ ($5.2x10^8$)	$1.64x10^9$ ($1.0x10^9$)	ns	0.160	133.2 (14.3)	136.7 (8.4)	ns	0.558
Mean (lEV+sEV)	$1.7x10^9$ ($1.3x10^9$)	$3.6x10^9$ ($4.5x10^9$)	ns	0.176	145.5 (24.1)	141.9 (11.1)	ns	0.555

Supplementary table S2

Surface protein analysis of certain EV associated markers compared between AM and PM as mean and 95% CI.

**Changes in EV surface markers**								
	**CD9 Response**				**CD81 Response**			
	**AM**	**PM**	**Difference**	***p***	**AM**	**PM**	**Difference**	***p***
Mean	4.27	3.81			6.50	6.09		
95% CI Lower	3.16	2.80	***	<.001	5.75	5.58	*	0.020
95% CI Upper	5.38	4.82			7.24	6.60		
	**CD8a Response**				**HLA-DR/DP/DQ Response**			
	**AM**	**PM**	**Difference**	***p***	**AM**	**PM**	**Difference**	***p***
Mean	2.30	1.89			1.94	1.65		
95% CI Lower	1.81	1.33	*	0.015	0.01	-0.81	*	0.029
95% CI Upper	2.79	2.46			3.88	3.38		

Supplementary table S3

Top 10 detected proteins in large and small EVs sorted by log FC.

Uniprot ID	Gene names	Protein Name	Difference (log FC)	*p*-value	*q*-value
**Proteins Upregulated in lEV**					
P21333	FLNA	Filamin-A	9.4	<0.0001	<0.0001
P35579	MYH9	Myosin-9	9.2	<0.0001	<0.0001
P18206	VCL	Vinculin	9.2	<0.0001	<0.0001
Q9Y490	TLN1	Talin-1	9.1	<0.0001	<0.0001
P12814	ACTN1	Alpha-actinin-1	8.6	<0.0001	<0.0001
Q9H4B7	TUBB1	Tubulin beta-1 chain	7.3	<0.0001	0.0013
P02730	SLC4A1	Band 3 anion transport protein	7.0	<0.0001	<0.0001
Q86UX7	FERMT3	Fermitin family homolog 3	6.9	<0.0001	<0.0001
P05106	ITGB3	Integrin beta-3	6.6	<0.0001	<0.0001
O75083	WDR1	WD repeat-containing protein 1	6.2	0.006	0.022
**Proteins Downregulated in lEV**					
**Uniprot ID**	**Gene names**	**Protein Name**	**Difference (log FC)**	***p*-value**	***q*-value**
P04275	VWF	von Willebrand factor	-2.3	<0.0001	<0.0001
P08519	LPA	Apolipoprotein(a)	-1.6	<0.0001	<0.0001
Q8WWA0	ITLN1	Intelectin-1	-1.4	<0.0001	<0.0001
P02753	RBP4	Retinol-binding protein 4	-1.3	<0.001	0.001
P02750	LRG1	Leucine-rich alpha-2-glycoprotein	-1.1	0.008	0.029
Q8WWU7	ITLN2	Intelectin-2	-1.0	<0.001	0.013
P19652	ORM2	Alpha-1-acid glycoprotein 2	-1.0	<0.001	0.012
P02763	ORM1	Alpha-1-acid glycoprotein 1	-0.9	<0.001	0.007
P06727	APOA4	Apolipoprotein A-IV	-0.9	<0.0001	<0.001
P01782	IGHV3-9	Immunoglobulin heavy variable 3-9	-0.8	0.001	0.012
